# Review of glioblastoma and systemic comorbidities

**DOI:** 10.3389/fonc.2026.1882982

**Published:** 2026-08-10

**Authors:** Zhang Tianqing, Guo Yuan, Yu Qianyi, Li Yanchu

**Affiliations:** 1 Outpatient Department, West China Hospital of Sichuan University, Chengdu, China; 2 Nursing Department, West China Hospital of Sichuan University, Chengdu, China; 3 Head & Neck Oncology Ward, West China Hospital of Sichuan University, Chengdu, China

**Keywords:** cognitive reserve, comorbidity networks, glioblastoma, health disparities, pharmacological interactions

## Abstract

Glioblastoma (GBM) is a highly aggressive malignancy with a poor prognosis. Despite advanced molecular characterization, the traditional tumor-centric model ignores the patient’s systemic body condition, which significantly influences disease progression and therapeutic outcomes. This review aims to elucidate the interactions between GBM and systemic comorbidities, focusing on metabolic, immune, and vascular networks. A key feature is activity-dependent gliomagenesis, in which neuronal firing via Neuroligin-3 (NLGN3) and glutamatergic signaling fuels tumor growth. This process confers a substantial cognitive reserve, thereby attenuating or masking early clinical manifestations via functional compensatory mechanisms; however, it may concomitantly promote rapid tumor proliferation. Overall, combined diabetes, glutamine-induced and hyperglycemia or dexamethasone-induced hyperglycemia may trigger tumor aggressive. The prothrombotic nature of GBM complicates the use of anti-angiogenic therapies. Thus, GBM and its comorbidities can be seen as metabolic-immune and vascular-cardiovascular cycles. Furthermore, chronic Hepatitis B (HBV) infection poses a unique risk of fatal reactivation during therapy in endemic regions, such as China. Consequently, real-world GBM management requires a shift from an isolated oncological to a patient-centric approach. Addressing systemic health disparities and the intrinsic interplay of comorbidities is essential to optimize treatment protocols and improve patient survival.

## Introduction

Glioblastoma (GBM) is the most prevalent and aggressive primary malignant brain tumor in adults (1). Despite the widespread adoption of the standard-of-care Stupp protocol, the median overall survival (OS) remains approximately 14–16 months. Over the past decade, the advent of high-throughput multiomics has meticulously mapped the genomic and transcriptomic heterogeneity of GBM, identifying critical prognostic markers, such as IDH mutation status and MGMT promoter methylation. However, the translation of these molecular targets into durable clinical therapies has faltered.

A critical flaw in contemporary neuro-oncology is the over-reliance on a tumor-centric model that evaluates malignancy in an isolated biological vacuum. GBM do not exist independently in real-world clinical settings. Patients have a complex, pre-existing physiological baggage of systemic comorbidities, varied cognitive reserves, and distinct sociodemographic backgrounds. Furthermore, the aggressive nature of the GBM treatment induces a cascade of secondary morbidities.

Epidemiological data from large-scale national registries have revealed counterintuitive phenomena. Higher educational attainment, which is an indirect indicator of intelligence quotient (IQ), and rigorous cognitive demands are positively correlated with the incidence of high-grade gliomas (2, 3). Initially dismissed by researchers as a mere detection bias; assuming that affluent, highly educated individuals had better access to neuroimaging-previous studies, suggested a biological link, such as higher education associated with socioeconomic factors, all the factors influencing the incidence of gliomas (4).

This review aimed to elucidate the dynamic interactions of host-derived factors in shaping the gliomagenesis. This study decoded the intrinsic logical relationships among these comorbidities to explain frequent clustering and collective compromise of therapeutic efficacy and patient survival.

### Literature search method

We searched MEDLINE via PubMed and Google Scholar for relevant preclinical and clinical studies, with no restrictions on publication year, study design, or sample type. The search combined MeSH terms and keywords related to glioma/GBM and comorbid conditions, such as “comorbidities/glioma”, “comorbidities/GBM”, “diabetes/glioma”, “hypertension/glioma”, “allergy/glioma”, “hepatitis B/glioma”, “epilepsy or epileptic seizures/glioma”, “cognitive reserve/glioma”, and “immune/glioma”. Titles and abstracts were independently screened for relevance, followed by a full-text assessment of potentially eligible articles. Studies were included if they addressed associations between gliomas or GBM and neurological, metabolic, immune, infectious, vascular, cardiovascular, or pharmacological comorbidities or provided mechanistic insight into these relationships. Articles unrelated to glioma/GBM comorbidities were excluded. In synthesizing the evidence, systematic reviews, meta-analyses, large cohort studies, randomized or prospective clinical studies, and well-designed translational investigations were prioritized. Smaller retrospective studies, case series, and preclinical studies were included if higher-level evidence was unavailable or they provided important mechanistic context.

## Results

### GBM and the activity-dependent tumor microenvironment

Historically, the GBM tumor microenvironment (TME) has been conceptualized as a battleground for immune evasion and metabolic competition (7). However, key findings in brain cancer research have shown that GBM is an electrically active entity that integrates into the patient’s neural network physically and functionally. This phenomenon, termed activity-dependent gliomagenesis, dictates that normal neuronal firing actively orchestrates tumor progression (**Table 1**).

**Table 1:** Impact of major systemic comorbidities on glioblastoma.

Comorbidity/host factor	Prevalence in GBM	Proposed mechanism	Impact on OS/risk	Clinical relevance	Evidence type & strength
DM	10% –15% (Global)	Metabolic Rewiring (Warburg Fuel): Chronic hyperinsulinemia-IGF1R upregulation-PI3K/Akt/mTOR activation	Reduced OS: (HR ~1.3-1.7 in DM vs. non-DM cohorts)	Hyperglycemia: Exacerbated by necessary Dexamethasone use (5); requires stringent glycemic control.	Preclinical mechanisms and clinical observations
Hypertension	20% – 30% (Age-dependent)	Vascular Paradox: General endothelial dysfunction; Bev-induced NO depletion-vasoconstriction.	Impact on OS: Primarily impacts Bev tolerance rather than direct OS linkage.	Stroke/ICH Risk: Balance anticoagulation against catastrophic hemorrhage on Bev therapy.	Emerging hypotheses
Allergy/Atopy	10%–15% (History of asthma, eczema, hay fever)	IgE-Mediated immunosurveillance: Heightened Th2-skewed immune vigilance eliminates early dysplastic glial cells.	Reduced Risk: (OR ~0.6-0.7 for atopy vs. no atopy) Improved OS	Potential therapeutic avenue through understanding immune vigilance mechanisms.	Emerging hypotheses
HBV (6)	Chinese Specificity: ~10% (HBsAg+ prevalence)	Iatrogenic Viral Activation: Dexamethasone + TMZ induces severe T-cell depletion and directly activates HBV GRE element.	Reduced OS: Associated with increased hepatic toxicity and TMZ dose reduction.	Hepatic Failure: Prophylactic antiviral therapy and stringent viral load monitoring are mandatory in endemic regions.	Clinical observations
Epileptic seizures	30%–50%	Hypoxia, mechanical compression, and glutamate release. Strongly correlated with IDH mutation.	Improved OS: Associated with earlier diagnosis and favorable IDH Status.	Drug-Drug Interactions: Enzyme-Inducing AEDs accelerate TMZ/Bev clearance via CYP450 system.	Preclinical mechanisms

DM, Diabetes Mellitus; OS, overall survival; HR, hazard ratio; Bev, bevacizumab; NO, nitric oxide; ICH, intracranial hemorrhage; OR, odds ratio; TMZ, temozolomide; GRE, glucocorticoid-responsive elements; AED, anti-epileptic drugs; IDH, isocitrate dehydrogenase; CYP450, cytochromeP450.

**Figure 1:**
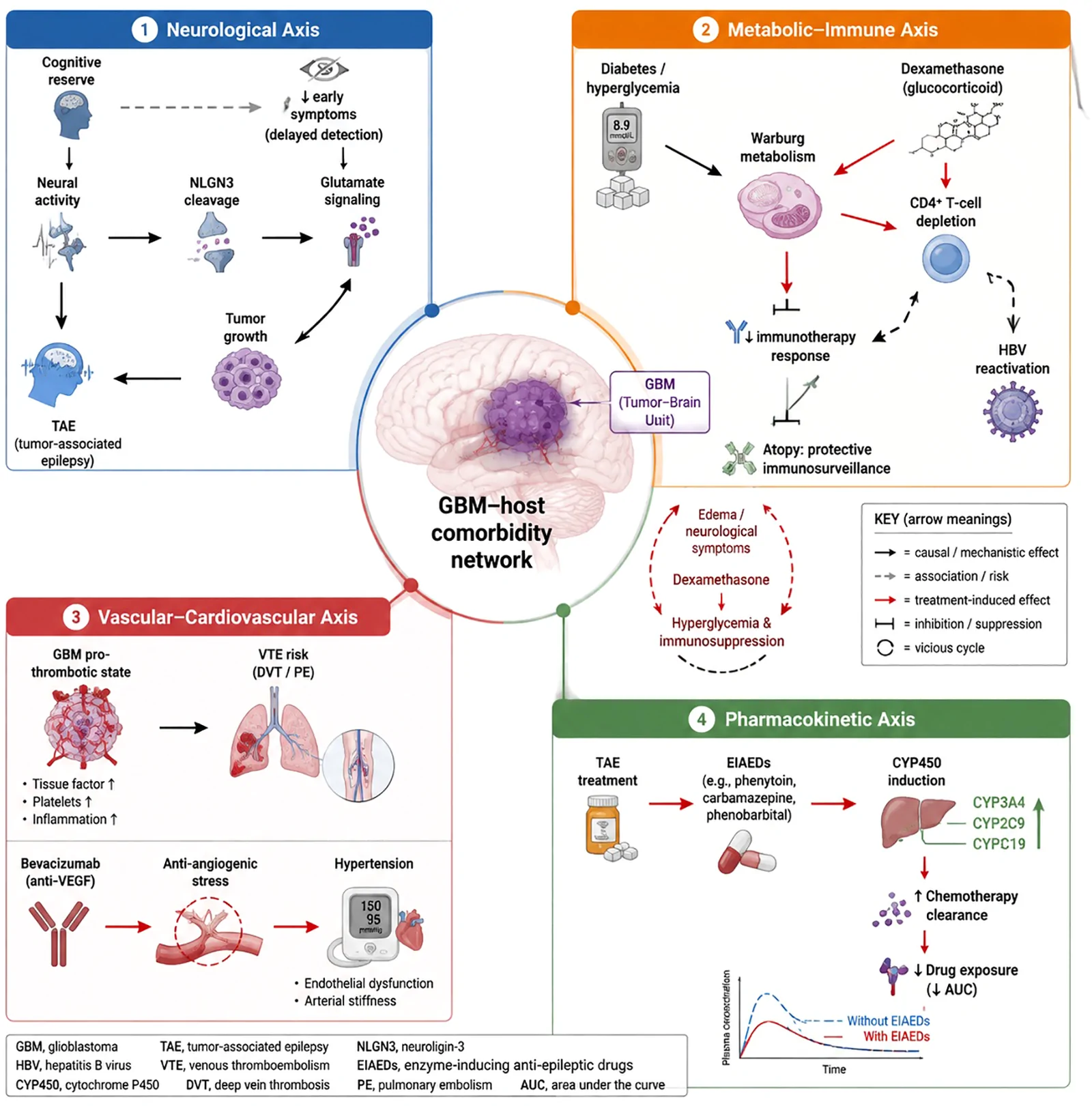
A systemic and microenvironmental conceptual panorama of GBM. Neurological Axis: A close-up synapse illustrates how Chronic Cognitive & Synaptic Hyperactivity triggers ADAM10-mediated cleavage of NLGN3, which fuels two pathways in GBM, show as PI3K/mTOR/Proliferation and Src Pathway/MMP-2 Up-regulation/Invasion. Meanwhile, neuron-Glioma Synapses (AMPA) and an extended network connected by Cx43 Gap Junctions, propagating Inter-tumoral Ca2^+^ Waves. Metabolic-Immune Axis: Dexamethasone acts as a master iatrogenic regulator, fueling the Warburg Effect and causing profound CD4+ T Cell depletion via Caspase-3 apoptosis. In endemic regions, this suppression directly triggers potentially fatal HBV reactivation, indicated as a region-specific clinical challenge. Pre-existing atopy (IgE-mediated) is shown to exert a protective Th2-skewed immunosurveillance effect against gliomagenesis. Vascular-Cardiovascular Axis: Bevacizumab controls edema but induces systemic NO Depletion/Vasoconstriction/Intractable Hypertension. Tumor-derived pro-thrombotic factors increase VTE risk, creating a vascular balancing act. Simultaneously, enzyme-inducing AEDs such as Phenytoin, hyperactivate the hepatic CYP450 enzyme system, leading to accelerated drug clearance and Sub-therapeutic drug levels, competing with Stupp protocol efficacy. Pharmacokinetic Axis: Tumor-associated epilepsy, enzyme-inducing anti-epileptic drugs such as phenytoin and carbamazepine can hyperactivate hepatic CYP450 pathways, accelerating the clearance of key oncologic agents and reducing therapeutic exposure. This drug–drug interaction creates an iatrogenic vulnerability in which seizure control may compromise the efficacy of standard GBM treatment, underscoring the need for non–enzyme-inducing AED selection and pharmacologic monitoring.

The core of this neuro-oncological crosstalk is the synaptic protein Neuroligin-3 (NLGN3) (8, 9). High cognitive demands and chronic neuronal activity in the peritumoral cortex trigger the activity-dependent cleavage of NLGN3 from presynaptic terminals, a process mediated by the metalloproteinase ADAM10 (**Figure 1**). Once secreted into the synaptic cleft, the soluble extracellular domain of NLGN3 binds to its corresponding receptor in glioma cells and acts as a potent mitogen. This paracrine signaling initiates a bifurcated oncogenic cascade which hyperactivates the PI3K/mTOR pathway to drive explosive cellular proliferation and upregulates the Src/MMP-2 axis to facilitate deep parenchymal invasion (10, 11). Moreover, GBM cells establish electrochemical connections with healthy host neurons. Recent ultrastructural and electrophysiological studies have revealed the formation of Neuron-Glioma Glutamatergic Synapses. Tumor cells receive direct excitatory synaptic inputs from the host network via functional AMPA-type glutamate receptors located on glioma cell membranes (12). Furthermore, individual glioma cells form extensive interconnected syncytia coupled with connexin 43 (Cx43) gap junctions (**Figure 1**) (13, 14). Excitatory inputs from a single neuron-glioma synapse can propagate across this malignant network via synchronous intertumoral calcium (*Ca*^2+^) waves, effectively amplifying the proliferative signal across the entire tumor bulk. This microenvironmental reality leads to a cognitive contradiction. A patient’s cognitive reserve, which is the baseline structural and functional robustness of the brain associated with better neurological outcomes, is a double-edged sword. Thus, high cognitive engagement and robust neuronal firing may directly fuel the ADAM10/NLGN3 axis and glutamatergic stimulation, paradoxically accelerating tumor growth (15–17).

Cognitive reserve is defined as the brain’s adaptability and capacity to functionally compensate for structural damage through pre-existing robust neural networks and universally viewed as a protective buffer (18, 19). However, if juxtaposed with the activity-dependent TME, a high cognitive reserve creates a clinical contradiction in neuro-oncology (20, 21). The intrinsic neuroplasticity and network redundancy that characterize high cognitive reserve effectively masks the initial space-occupying and destructive effects of GBM. Functional reorganization allows the host brain to reroute critical cognitive and motor commands around expanding lesions. Consequently, although this is currently only a hypothesis, patients with robust cognitive reserve often remain asymptomatic or present with only vague, non-specific symptoms during the early stages of glioma genesis (22).

This functional compensation may lead to illness neglect. If the tumor burden overwhelms the brain’s compensatory capacity and elicits definitive focal neurological deficits, GBM often achieves a massive volume and deeply infiltrates bilateral structures. Thus, it may obscure the window of opportunity for early surgical intervention and the neuronal activity required to sustain this functional compensation continuously fuels tumor proliferation and invasion via the NLGN3 and glutamatergic axes (**Figure 1**). The highly connected brain unknowingly nourishes the parasite it is attempting to work around. The tipping point of this prolonged “silent” progression frequently manifests as a catastrophic systemic event, most notably, first-onset tumor-associated epilepsy (TAE). As the expanding tumor mass compresses the adjacent healthy parenchyma and local microenvironment becomes saturated with glutamate, which is exacerbated by excitatory amino acid transporter 2 (EAAT2) downregulation in reactive astrocytes, the epileptogenic threshold is breached (23, 24).

Furthermore, this localized neuro-excitability reaches pathological thresholds, manifesting macroscopically as TAE (25, 26). According to data from the Chinese Glioma Genome Atlas (CGGA), this hyperexcitable phenotype is enriched in secondary GBM harboring IDH1/2 mutations, in which the oncometabolite D-2-hydroxyglutarate (D-2-HG) structurally mimics glutamate, persistently agonizes NMDA receptors, and lowers the seizure threshold (27, 28). Therefore, TAE management is an intervention in the tumor’s fundamental growth mechanism. However, the use of anti-epileptic drugs (AEDs) to suppress this network forces clinicians to navigate complex sets of interactions throughout the body. The selection of AEDs dictates whether the patient falls into a vicious iatrogenic cycle of accelerated chemotherapeutic clearance or successfully preserves the efficacy of the Stupp protocol.

### Comorbidity axis of GBM

A patient’s systemic physiological health exerts immense pressure on GBM progression. Systemic factors include metabolic, vascular, and immune factors (**Table 1**).

Firstly, in the metabolic axis, pre-existing diabetes mellitus (DM) and perioperative hyperglycemia have emerged as robust, independent prognosticators of reduced survival in GBM (29–31). This relationship extends beyond the increased risk of surgical site infections and delayed wound healing. Chronic hyperinsulinemia and elevated systemic glucose levels actively fuel metabolic rewiring of cancer cells, exacerbating the Warburg effect (32, 33). Moreover, high circulating insulin and glucose levels upregulate insulin-like growth factor-1 (IGF-1) receptor signaling on the tumor cell surface, hyperactivating pro-survival PI3K/Akt/mTOR cascades (34, 35). Although repurposing anti-diabetic agents, such as metformin, which activates AMPK to inhibit mTOR and potentially targets glioma stem cells (GSCs), demonstrate synergistic anti-neoplastic potential in preclinical models, retrospective clinical data remain conflicting (36, 37). This underscores the complexity of treating metabolic dysregulation once the tumor architecture is fully established.

Meanwhile, beyond diabetes, hyperglycemia, and dexamethasone-induced metabolic disturbances as clinically relevant variables in glioma, increasing experimental evidence suggests that glutamine also serves as a critical substrate for glioblastoma bioenergetics, redox homeostasis, and biosynthesis (38). In this context, discussion of glutaminolysis, glutamate production, and mitochondrial metabolism is warranted, as these interconnected pathways may provide a more comprehensive framework for understanding the metabolic network that underlies glioblastoma progression. Integrating these processes with glucose-related metabolic alterations may therefore help refine the current view of glioblastoma metabolic programming. Mechanistically, glutamine metabolism in glioblastoma extends beyond a simple auxiliary nutrient pathway and instead constitutes a central metabolic axis linking carbon and nitrogen supply, mitochondrial anaplerosis, redox buffering, and microenvironmental remodeling. After cellular uptake, glutamine can be converted by glutaminase to glutamate, which is subsequently directed toward α-ketoglutarate production through glutamate dehydrogenase or transaminase-dependent reactions, thereby replenishing the tricarboxylic acid cycle and supporting oxidative metabolism as well as biosynthetic demand (39). Beyond its role as a mitochondrial fuel, glutamate also contributes to glutathione synthesis and thus helps sustain redox homeostasis under metabolic stress. In addition, BCAT1-associated transamination may further reinforce glutamate-centered nitrogen metabolism, particularly in IDH-wild-type glioma, and has been linked to aggressive and poorly differentiated tumor phenotypes (40). At the same time, dysregulated glutamate handling, including xCT-associated glutamate export, may reshape the tumor microenvironment and promote glioma growth, invasion, and excitotoxic injury in surrounding neural sue (41, 42). Thus, glioblastoma metabolic programming may influence tumor aggressiveness.

Moreover, when glioma patients receive radiation, ionizing radiation induces astrocytic reactivity, neuroinflammation, oxidative stress, mitochondrial dysfunction, and cellular senescence, all of which can remodel the irradiated milieu in ways associated with recurrent glioblastoma aggressiveness (43). Given that astrocytes are central to glutamate clearance and glutamine recycling in the brain, and that γ-irradiation alters glutamate transporter expression and uptake in neurons and astrocytes, radiotherapy may also disturb glutamine-glutamate homeostasis (44). In parallel, extracellular glutamate is already known to promote glioma-associated neuronal injury, peritumoral hyperexcitability, and tumor growth (45). Taken together, radiotherapy may inadvertently generate metabolic conditions favorable for surviving glioblastoma cells, although direct evidence that this occurs specifically through disruption of glutamine-glutamate cycling remains limited (46).

Although glutamate dehydrogenase- or transaminase-mediated reactions generate α-ketoglutarate (α-KG), thereby replenishing the tricarboxylic acid (TCA) cycle and supporting oxidative metabolism and biosynthetic processes, however, under hypoxic conditions, glutamine-driven mitochondrial substrate-level phosphorylation (mSLP) may provide an alternative mechanism for maintaining ATP production in glioblastoma (GBM) cells. Chinopoulos et al. proposed in 2018 that glutamine-derived α-KG can be metabolized through the α-KG dehydrogenase complex–succinyl-CoA ligase (KGDHC–SUCL) segment of the TCA cycle. This pathway enables the direct formation of ATP or GTP in the mitochondrial matrix through the succinyl-CoA ligase reaction, thereby providing an energy source that does not require molecular oxygen to serve as the terminal electron acceptor (47). More recent work by Derek C. Lee et al. in 2024 further demonstrated that glutamine can sustain ATP levels in both murine and human glioma cells independently of oxygen availability. ATP levels were maintained under hypoxic and glucose-deprived conditions, whereas pharmacological inhibition of glutaminase decreased cellular ATP levels (48). Together, these findings support the conclusion that glutamine-fueled mSLP in the mitochondrial matrix can generate a substantial amount of oxygen-independent ATP and thereby contribute to the growth and survival of GBM cells.

Collectively, glioblastoma is better understood as a disease developing within an integrated host environment. Many of the comorbidities and modifying factors discussed above, including diabetes, immune dysfunction, thrombosis, altered neuronal activity, and treatment-related toxicities, ultimately converge on perturbations in energy metabolism. In this sense, energy metabolism may represent a common denominator through which systemic conditions, microenvironmental stressors, and therapeutic interventions collectively shape glioblastoma progression. Thus, these findings suggest that therapeutic interventions should be considered not only for their cytotoxic effects on tumor cells, but also for their capacity to reshape the local and systemic metabolic environment in ways that influence disease progression (49).

Secondly, the vascular axis includes thrombosis and angiogenesis. GBM inherently foster a prothrombotic milieu. Tumor cells systemically release tissue factor (TF) and procoagulant microvesicles, resulting in the highest rates of venous thromboembolism (VTE) and deep vein thrombosis (DVT) among all solid malignancies, affecting approximately 20% of patients (50–52). Thus, baseline hypercoagulability creates a formidable vascular paradox if initiating anti-angiogenic therapies, most notably bevacizumab, which is used for recurrent GBM. Although bevacizumab effectively prunes the abnormal tumor vasculature and mitigates life-threatening peritumoral edema, systemic VEGF inhibition causes widespread endothelial dysfunction. This leads to intractable, treatment-emergent hypertension and an elevated risk of arterial thromboembolic events (ATEs) (53). Balancing systemic anticoagulation for VTE against the catastrophic risk of spontaneous intracranial hemorrhage (ICH) is among the most perilous challenges in neuro-oncology (54).

Thirdly, a compelling inverse association has been consistently documented between atopic conditions and GBM risk (55, 56). Large epidemiological cohorts have revealed that individuals with a history of allergies have a 30–40% reduced risk of developing gliomas (57–59). This “atopy-protective effect” suggests that a hyper-responsive immune system, characterized by elevated serum immunoglobulin E (IgE) and a Th2-skewed inflammatory profile, may exert heightened immunosurveillance (60, 61). Hypervigilant state enables the immune system to recognize and eliminate early-stage dysplastic glial cells before establishing an immunosuppressive TME. Deciphering the specific IgE-mediated cytotoxicity against glioma antigens remains a promising frontier for developing novel immunotherapies.

### GBM and the intrinsic interplay of comorbidities

Comorbidities in GBM do not operate in isolated silos and are deeply interconnected through shared pathophysiological mechanisms and iatrogenic triggers. To understand these relationships, we discussed two possible cycles driving the clustering of comorbidity in GBM (**Table 1**).

The iatrogenic–metabolic–immune cycle; the most prominent intrinsic relationship is driven by dexamethasone, which is used to control peritumoral edema. Dexamethasone possesses a profound glucocorticoid activity that rapidly induces hepatic gluconeogenesis and peripheral insulin resistance, precipitating severe iatrogenic hyperglycemia. Thus, this hyperglycemia fuels tumor metabolism and synergizes with dexamethasone to induce profound systemic immunosuppression (5, 62). Previously, higher doses may be beneficial pre- and postoperatively but detrimental during adjuvant therapy (63). Dexamethasone directly causes apoptosis and depletion of peripheral CD4+ T lymphocytes and downregulates MHC expression in antigen-presenting cells (64). Consequently, immunoparalysis renders the patient highly susceptible to opportunistic infections, such as mycotic pneumonia. Insidiously, this immunosuppression creates an environment in which tumors can completely evade immune checkpoint blockade therapies, rendering them ineffective. Thus, attempts to treat neurological symptoms, such as edema, directly worsen systemic metabolism and suppress systemic immunity.

Conversely, the vascular-cardiovascular axis; the second intrinsic relationship connects tumor vascularity, cardiovascular health, and epileptogenesis. For the mechanism, the rapid, disorganized angiogenesis of GBM creates regions of severe hypoxia. Treatments, such as bevacizumab, aim to normalize this but often induce systemic hypertension and further local microvascular ischemia. Thus, hypoxia and microvascular ischemia in the surrounding brain parenchyma lowers the depolarization threshold of adjacent healthy neurons. This hypoxic stress, combined with mechanical compression by the tumor and neurotoxic release of glutamate via the xc-transporter system by tumor cells, creates a highly excitatory microenvironment, leading to TAE. Therefore, AEDs can be used (65). According to the pharmacokinetic axis, traditional enzyme-inducing AEDs (EIAEDs), such as phenytoin or carbamazepine, hyperactivate the hepatic Cytochrome P450 (CYP450) enzyme system (66). This induction rapidly accelerates the clearance of cardiovascular drugs, such as the antihypertensives needed for bevacizumab-induced hypertension, and chemotherapeutics, such as TMZ, leading to sub-therapeutic drug levels. Therefore, vascular pathology drives neurological comorbidities, which disrupt the pharmacological management of tumors and systemic diseases via complex drug-drug interactions (DDIs).

### Glioblastoma and comorbidities in real-world

The physiological interplay of comorbidities is significantly exacerbated by macro-level sociodemographic, racial, and geographic disparities, which extend beyond the underlying genomic heterogeneity. Registry data consistently demonstrate that although non-Hispanic Whites exhibit a higher baseline incidence of GBM, minority populations often experience inferior real-world clinical outcomes (67) driven by a disproportionate baseline burden of poorly controlled systemic comorbidities, such as severe hypertension (68, 69), obesity (70), and diabetes (29, 71), during initial GBM diagnosis. This pre-existing systemic health disparity serves as a barrier to standard-of-care management. Patients with high comorbidity burden have lower physiological resilience, resulting in higher rates of therapeutic attrition. Patients frequently suffer from unbearable hematological or organ toxicities during concurrent chemoradiotherapy, necessitating premature dose reduction or complete cessation of the Stupp protocol, which directly reduces overall survival.

Insights from Chinese cohorts, extensive data from the CGGA, and large Chinese clinical cohorts offer region-specific perspectives (72). Patients residing in rural areas face significant logistical and socioeconomic barriers in accessing specialized neuro-oncological centers. The Stupp protocol mandates six weeks of continuous daily radiotherapy, and rural patients experience severe disruptions to this schedule because of travel constraints and lack of local supportive care for managing concurrent morbidities, significantly compromising local tumor control. A critical, often overlooked comorbidity unique to endemic regions is chronic HBV infection. This interplay exemplifies the biological networks discussed earlier. The concomitant use of TMZ and high-dose dexamethasone places patients with HBV-carrying GBM at an exceptionally high risk of fulminant hepatic failure secondary to fatal viral reactivation (73). This mechanism is two-fold. Dexamethasone induces profound systemic T cell depletion and directly binds to glucocorticoid-responsive elements (GRE) within the HBV genome, actively stimulating viral transcription. Consequently, Chinese consensus guidelines mandate prophylactic antiviral therapy, such as entecavir, and stringent HBV DNA viral load monitoring throughout the neuro-oncology treatment course, a nuance frequently absent in Western guidelines but crucial for patient survival in the Asia-Pacific region.

### Whole-process management in supportive care strategies for GBM

The clinical implications of whole-process management in supportive care extend beyond tumor-directed therapy alone and support the integration of multimodal supportive care throughout the glioblastoma disease course. In practice, this includes nutritional support targeting altered glucose metabolism, structured physical activity and neurorehabilitation to preserve functional capacity, and proactive management of pain, seizures, cognitive decline, fatigue, and other neurological symptoms. Measures to reduce chronic systemic inflammation and catabolic stress may also help maintain treatment tolerance and quality of life. Importantly, this supportive care should be regarded not as adjuncts, but as integral components of care. At the same time, for patients with progressive neurological deterioration, poor performance status, or refractory disease, the potential benefit of continued aggressive therapy should be carefully weighed against cumulative toxicity and further loss of quality of life, with timely goals-of-care discussions to identify when symptom-directed, rehabilitative, and palliative-focused approaches may offer greater benefit.

## Conclusion

GBM is a systemic disease that causes intracranial hypertension and is associated with cognitive function, systemic metabolism, vascular health, and sociodemographic factors. These comorbidities are intrinsically linked via shared pathogenic mechanisms and iatrogenic pharmacological cascades, thereby creating a vicious cycle of immunosuppression, metabolic disorders, and neurotoxicity. Clinically, the following approaches are used: adjusting glucocorticoid dosage based on disease status, monitoring blood glucose levels during GBM treatment, conducting HBV screening and preventive antiviral therapy in endemic regions, prioritizing non-enzyme-inducing anti-epileptic drugs, assessing and managing the risk of VTE, and multidisciplinary team management, which may serve as a treatment option for gliomas and their complications, but it is too early to draw definitive conclusions.
